# Projected changes in climatic suitability for *Kinosternon* turtles by 2050 and 2070

**DOI:** 10.1002/ece3.2492

**Published:** 2016-10-05

**Authors:** Christopher J. Butler, Brian D. Stanila, John B. Iverson, Paul A. Stone, Matthew Bryson

**Affiliations:** ^1^ Department of Biology University of Central Oklahoma Edmond OK USA; ^2^ Department of Biology Earlham College Richmond IN USA

**Keywords:** chelonian, climate change, distribution, ecological niche model, Maxent, turtle

## Abstract

Chelonians are expected to be negatively impacted by climate change due to limited vagility and temperature‐dependent sex determination. However, few studies have examined how freshwater turtle distributions may shift under different climate change scenarios. We used a maximum entropy approach to model the distribution of five widespread North American *Kinosternon* species (*K. baurii*,* K. flavescens*,* K. hirtipes*,* K. sonoriense*, and *K. subrubrum*) under four climate change scenarios. We found that areas with suitable climatic conditions for *K. baurii* and *K. hirtipes* are expected to decline substantially during the 21st century. In contrast, the area with suitable climate for *K. sonoriense* will remain essentially unchanged, while areas suitable for *K. flavescens* and *K. subrubrum* are expected to substantially increase. The centroid for the distribution of four of the five species shifted northward, while the centroid for *K. sonoriense* shifted slightly southward. Overall, centroids shifted at a median rate of 37.5 km per decade across all scenarios. Given the limited dispersal ability of turtles, it appears unlikely that range shifts will occur rapidly enough to keep pace with climate change during the 21st century. The ability of chelonians to modify behavioral and physiological responses in response to unfavorable conditions may allow turtles to persist for a time in areas that have become increasingly unsuitable, but this plasticity will likely only delay local extinctions.

## 1 Introduction

Many studies have linked changes in the distribution and phenology of multiple organisms to anthropogenic climate change (e.g., Hughes, 2000; Lafferty, 2009). For example, numerous bird species began arriving earlier as the earth warmed by 0.6 ± 0.2°C during the 20th century (Butler, 2003; Cotton, 2003; Hurlbert & Liang, 2012) and several authors noted changes in distribution for numerous taxa (e.g., Parmesan & Yohe, 2003; Perry et al., 2005). In addition to causing phenological and distributional shifts, anthropogenic climate change increases the risk of extinction by reducing the amount of suitable habitat (Sekercioglu et al., 2008; Thuiller et al., 2006).

Because turtles have relatively limited dispersal capabilities (Gibbons et al., 2000) and often exhibit temperature‐dependent sex determination (Janzen, 1994), global climate change may have severe negative impacts on turtle populations. Specifically, climate change is predicted to affect individual growth rates (Du & Ji, 2003), population sex ratios (Janzen, 1994), fecundity (Ficetola, Thuiller, & Padoa‐Schioppa, 2009), reproductive phenology (Lovich et al., 2012), and predation rates (Chessman, 2011; Christiansen et al. 2012). Most studies examining how climate change may affect chelonians have focused on sea turtles (e.g., Hawkes et al., 2009; Rees et al., 2010), with relatively little attention given to freshwater turtles. Ihlow et al. (2012) used species distribution modeling to assess species richness of turtles on a global scale and to project future distributions based on climate change scenarios released by the IPCC (2007). Their analysis concluded that 86% of turtle species will experience range contractions due to climate change, with nearly 12% of these species predicted to experience range shifts so drastic that future geographic ranges will be completely outside existing ranges (Ihlow et al., 2012).

New World mud turtles in the genus *Kinosternon* are small, semi‐aquatic turtles that occupy a range of ecologically diverse habitats including ephemeral drainage ditches, intermittent canyon pools and streams, backwaters of slow‐moving rivers and lakes, and even estuaries (Ernst & Lovich, 2009). Ecological and physiological adaptations allow mud turtles to be successful in both aquatic and terrestrial environments facilitating their widespread distribution, which includes much of the New World (Ernst & Lovich, 2009; Iverson, Le, & Ingram, 2013).

Despite their widespread distribution, fossil examples of *Kinosternon* are relatively sparse (Bourque, 2012; Cadena, Jaramillo, & Paramo, 2007). The fossil record indicates that by the early to middle Miocene, *Kinosternon* existed across North America (Bourque, 2012, 2015; Holman, 1998; Joyce & Bourque, 2016). *Kinosternon* were present in Central America by at least the early Pleistocene (Cisneros, 2005), and South America by the late Pleistocene (Cadena et al., 2007). Many of the aforementioned fossils were discovered at locations outside current geographic ranges of *Kinosternon*, suggesting much wider distributions in the past. The genus *Kinosternon* likely has a long history of responding to climate change, as do other turtle species that occur in regions affected by glaciation (Rödder et al., 2013; Starkey et al., 2003). However, recent anthropogenic climate change is occurring far more rapidly than previous events (Intergovernmental Panel on Climate Change, 2014) and the effect that this may have on the distribution of *Kinosternon* remains unexamined.

Multiple modeling approaches exist for evaluating how the distribution of organisms may change through time (Bakkenes et al., 2002; Huntley et al., 1995; Matthews et al., 2011). Models that rely upon bioclimatic variables predict fundamental niches, as physiological constraints will limit organisms to a subset of values for those variables (Pearson & Dawson, 2003). In particular, a maximum entropy approach (Maxent) is especially suitable as it models the distribution of organisms using solely presence data rather than presence and absence (or pseudo‐absence) data (Phillips, Anderson, & Schapire, 2006; Phillips & Dudik, 2008). Maxent modeling is a common technique to predict the ranges of a large number of diverse taxa (Butler, Wheeler, & Stabler, 2012; Papes & Gaubert, 2007; Phillips & Dudik, 2008; Ward, 2007).

We used Maxent to model the niches of five widespread species of *Kinosternon* whose geographic ranges include the United States. We then modeled future niches under four climate change scenarios, in an attempt to anticipate the effects of climate change on their future geographic distributions.

## 2 Materials and Methods

**Figure 1:**
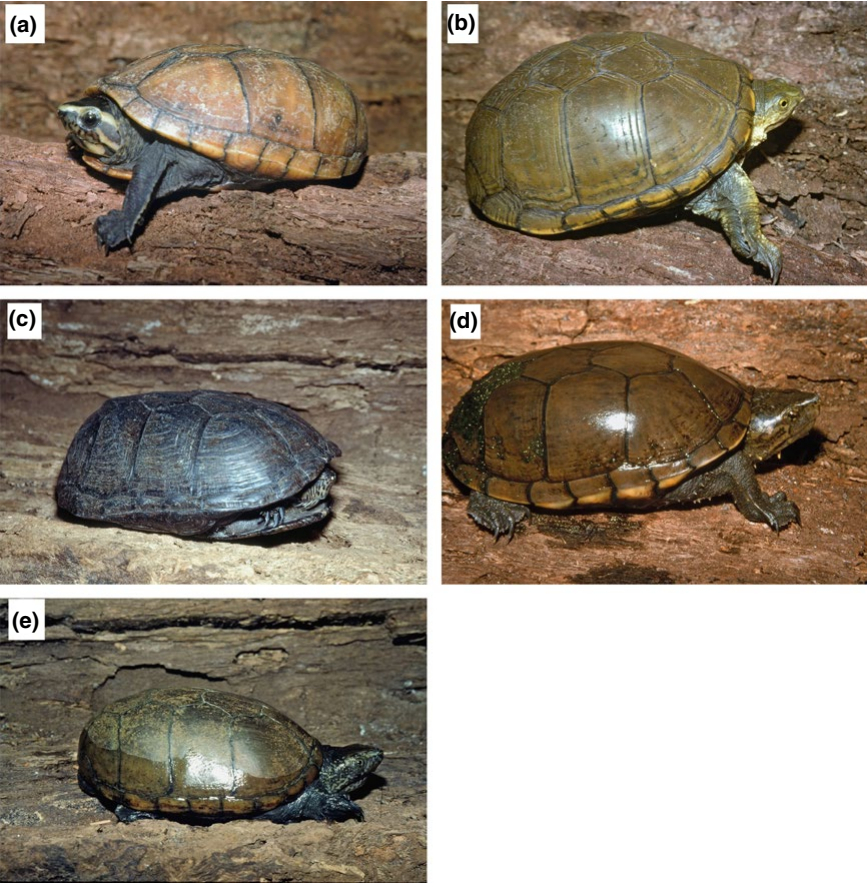
The five species included in this study are *Kinosternon baurii* (a), *Kinosternon flavescens* (b), *Kinosternon hirtipes* (c), *Kinosternon sonoriense* (d), and *Kinosternon subrubrum* (e)

We used Maxent to model the current and projected distribution of five widespread *Kinosternon* species: *K. baurii*,* K. flavescens, K. hirtipes, K. sonoriense*, and *K. subrubrum* (Phillips, Dudik, & Schapire, 2004; Phillips et al., 2006; Figure 1). We downloaded records of these five species from the EmySystem (Kiester & Bock, 2007), combined them with field observations of *K. sonoriense*, and cleaned these records for duplicates and errors (Newbold, 2010). Records of *K. arizonense* and *K. durangoense*, formerly regarded as subspecies of *K. flavescens* (Iverson et al., 2013; Serb, Phillips, & Iverson, 2001), were not included in the analysis. We resampled the locality data so that there was only one record per 25 km^2^ using ENMTools (Warren, Glor, & Turelli, 2010). We downloaded elevation and 19 bioclimatic variables from WorldClim (Hijmans et al., 2005; http://www.worldclim.org/) at a resolution of 2.5 arc minutes (25 km^2^; Table 1). We trimmed the spatial extent of the variables in ArcGIS to include the area from Mexico through the southern half of Canada (ESRI 2006). Initially, all variables were included in the model, but only the variables with the highest gain when used in isolation were retained, as these variables appeared to provide the most useful predictive information. In addition, the environmental variables that decreased the gain the most when they were omitted were also retained, as these variables appeared to provide unique predictive information. We then checked variables for high multicollinearity (|*r*| > 0.8; Jones, Acker, & Halperin, 2010). We avoided model overfitting using a regularization approach which introduced a penalty for an increase in model complexity (Merckx et al., 2011; Phillips et al., 2006), and the small sample corrected variant of Akaike's information criterion (AICc) scores was used to evaluate the regularization of models (Warren & Seifert, 2011) using all possible combinations of the variables that did not exhibit high multicollinearity. Receiver operating characteristic (ROC) curves were created by plotting sensitivity vs. specificity, and tenfold cross‐validation AUC (area under the curve) scores were used to evaluate the accuracy of the resulting model. Models with an AUC score of 0.5 indicate a model performing no better than random, while models with an AUC score of 1 indicate a perfect model (Phillips et al., 2004, 2006). AUC scores are not without limitations (for example, they are affected by the spatial extent of the area sampled; Lobo, Jiménez‐Valverde, & Real, 2008; Elith et al., 2011), and it has been recommended that they be used in conjunction with other methods of evaluating models (So & Sham, 2010). Consequently, we used AICc scores and model weights in conjunction with AUC scores to determine the models that best describe the current distributions of the five species of *Kinosternon*.Future climate conditions for 2050 and 2070 using the IPCC 5 data from WorldClim (Hijmans et al., 2005) were used to project the potential future distribution of *K. baurii*,* K. flavescens, K. hirtipes, K. sonoriense*, and *K. subrubrum* at 2.5 arc minutes (25 km^2^) using the model that best predicted the current distribution of each species. Four IPCC scenarios were evaluated: RCP 2.6 (characterized by carbon dioxide emissions peaking prior to 2020 and declining thereafter), RCP 4.5 (emissions peak around 2040 and then decline), RCP 6.0 (emissions peak around 2080 and then decline), and RCP 8.5 (emissions increase throughout the 21st century) using 11 different general circulation models downloaded from WorldClim (BCC‐CSM1‐1, CCSM4, GISS‐E2‐R, HadGEM2‐AO, HadGEM2‐ES, IPSL‐CM5A‐LR, MIROC‐ESM‐CHEM, MIROC‐ESM, MIROC5, MRI‐CGCM3, and NorESM1‐M). Model averaging was used to create models of projected suitability under each RCP scenario for 2050 and the 2070.

**Table 1:** Summary of bioclimatic variables used in this study

Variable	Definition
BIO 1	Annual mean temperature
BIO 2	Mean diurnal range (mean of monthly [max temp − min temp])
BIO 3	Isothermality (BIO 2 / BIO 7) × 100
BIO 4	Temperature seasonality (standard deviation × 100)
BIO 5	Max temperature of warmest month
BIO 6	Min temperature of coldest month
BIO 7	Temperature annual range (BIO 5–BIO 6)
BIO 8	Mean temperature of wettest quarter
BIO 9	Mean temperature of driest quarter
BIO 10	Mean temperature of warmest quarter
BIO 11	Mean temperature of coldest quarter
BIO 12	Annual precipitation
BIO 13	Precipitation of wettest month
BIO 14	Precipitation of driest month
BIO 15	Precipitation seasonality (coefficient of variation)
BIO 16	Precipitation of wettest quarter
BIO 17	Precipitation of driest quarter
BIO 18	Precipitation of warmest quarter
BIO 19	Precipitation of coldest quarter
Elevation	Elevation above sea level

## 3 Results

**Figure 2:**
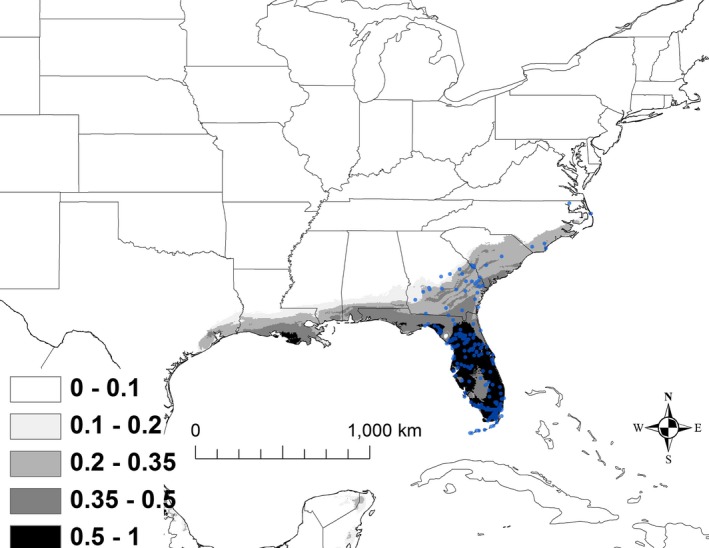
The modeled current distribution for *Kinosternon baurii*. The probability is shown in gray scale in the legend; the darkest shade shows an area with >0.5 probability of occurrence. Locations where *K. baurii* (*n = *211) were recorded as present based on EmySystem database and field observations are shown with blue dots

The best model for *K. baurii* (i.e., with the lowest AICc score) included the variables annual mean temperature (BIO 1), mean temperature of wettest quarter (BIO 8), precipitation of wettest quarter (BIO 16), and precipitation of warmest quarter (BIO 18; Table 2). The AUC for this model was 0.986 ± 0.004. Areas that were predicted to have suitability >50% had an annual mean temperature of 22–24°C, a mean temperature of the wettest quarter of 27–28°C, precipitation of the wettest quarter ranging from 51 to 64 cm, and precipitation of the warmest quarter ranging from 49 to 62 cm. Areas that are currently shown as >50% suitability were primarily restricted to Florida (Figure 2).

**Table 2:** A comparison of the top model runs for each species

Species	Variables	Log‐likelihood	AIC_c_ score	ΔAIC_c_	wAIC_c_	Mean AUC
*Kinosternon baurii*	BIO 1, BIO 8, BIO 16,BIO 18	−2,137.721	4,342.748	0	1	0.986
*Kinosternon flavescens*	BIO 8, BIO 9, BIO 10,BIO 15	−7,157.165	14,428.017	0	1	0.941
*Kinosternon hirtipes*	BIO 6, BIO 15, BIO 17,elevation	−1,320.249	2,719.672	0	0.423	0.983
	BIO 4, BIO 15, BIO 17,elevation	−1,320.749	2,720.673	1.001	0.256	0.983
	BIO 6, BIO 15, BIO 17	−1,335.399	2,721.515	1.842	0.168	0.981
	BIO 4, BIO 15,elevation	−1,334.259	2,722.157	2.484	0.122	0.982
*Kinosternon sonoriense*	BIO 2, BIO 4, BIO 8,BIO 14	−1,668.1444	3,420.533	0	0.590	0.976
	BIO 1, BIO 2, BIO 4,BIO 8, BIO 14	−1,660.954	3,423.026	2.493	0.170	0.975
	BIO 1, BIO 2, BIO 4,BIO 8, BIO 17	−1,642.117	3,423.790	3.257	0.116	0.976
	BIO 3, BIO 5, BIO 8,BIO 17	−1,673.281	3,424.434	3.901	0.084	0.973
*Kinosternon subrubrum*	BIO 6, BIO 10, BIO 12elevation	−15,855.610	31,797.221	0	1	0.915

Log‐likelihood is the natural log of the probability of the data given in the model. AICc is a corrected AIC score, used for a small sample size by increasing the cost for each parameter. Only models that are within four units of the top AIC model are shown. Delta AICc is the difference between the model with the lowest score (the “best” model) and the AICc score for each model. The model weight (wAICc) is the relative likelihood for each model, divided by the total relative likelihood for all models that were considered. AUC (area under the curve) is a measure of the accuracy of the model.

**Figure 3:**
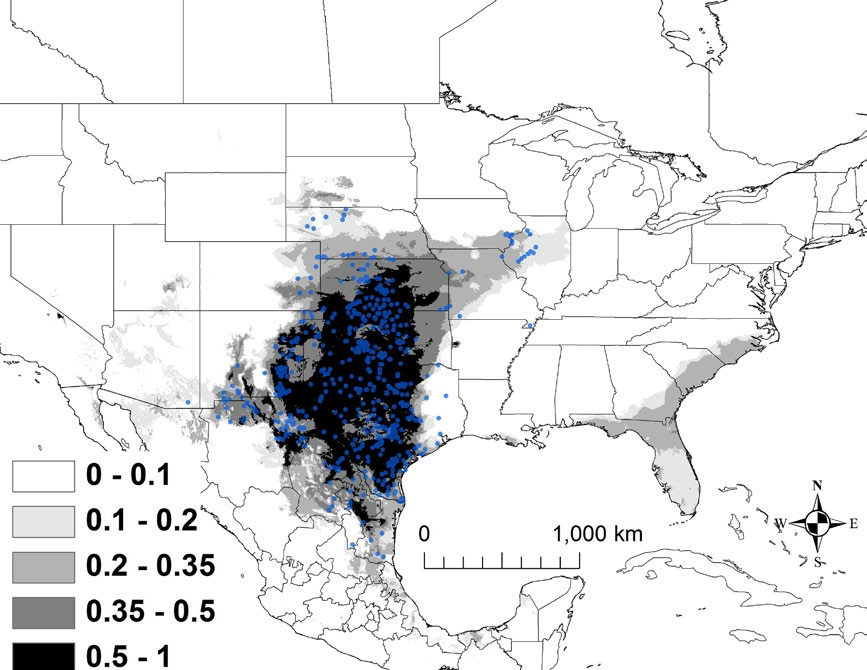
The modeled current distribution for *Kinosternon flavescens*. The probability is shown in gray scale in the legend; the darkest shade shows an area with >0.5 probability of occurrence. Locations where *K. flavescens* (*n = *627) were recorded as present based on EmySystem database and field observations are shown with blue dots

The best model for *K. flavescens* included mean temperature of wettest quarter (BIO 8), mean temperature of driest quarter (BIO 9), mean temperature of warmest quarter (BIO 10), and precipitation seasonality (BIO 15; Table 2). The AUC for this model was 0.941 ± 0.005. Areas that were predicted to have suitability >50% had a mean temperature of the wettest quarter of 22–30°C, a minimum temperature of the driest quarter of 1–10°C, a mean temperature of the warmest quarter of 26–30°C, and with a relatively wide range of precipitation seasonality (the coefficient of variation ranged from 31 to 112). Areas that are currently shown as >50% suitability extended from Tamaulipas north to Kansas and west to New Mexico and extreme northeastern Chihuahua (Figure 3).

**Figure 4:**
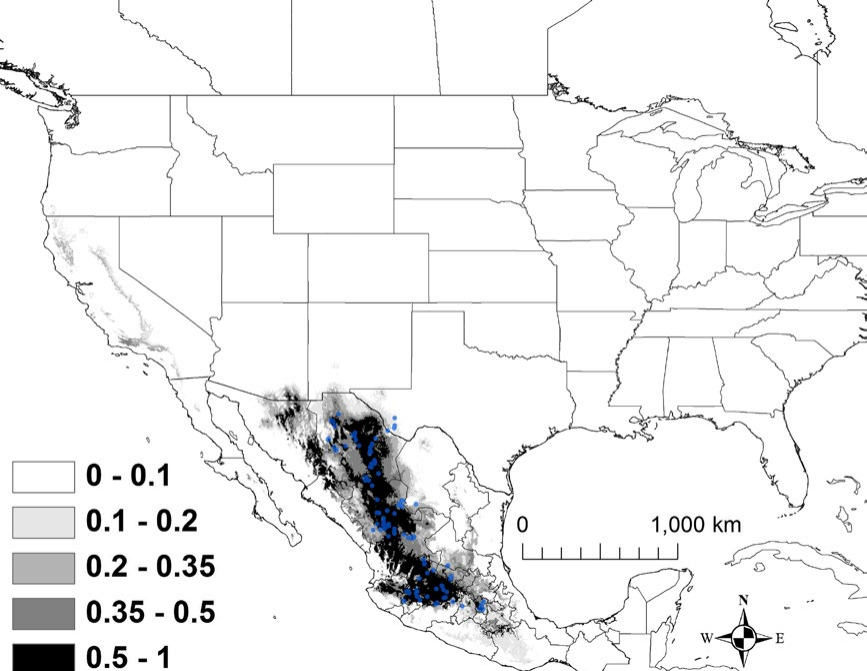
The modeled current distribution for *Kinosternon hirtipes*. The probability is shown in gray scale in the legend; the darkest shade shows an area with >0.5 probability of occurrence. Locations where *K. hirtipes* (*n = *129) were recorded as present based on EmySystem database and field observations are shown with blue dots

The best model for *K. hirtipes* included minimum temperature of the coldest month (BIO 6), precipitation seasonality (BIO 15), precipitation of driest quarter (BIO 17), and elevation (Table 2). The AUC for this model was 0.983 ± 0.004. There was also some model support for temperature seasonality (BIO 4; Table 2). Areas that were predicted to have suitability >50% had a minimum temperature of 1–8°C, a relatively narrow range of precipitation seasonality (the coefficient of variation ranged from 95 to 111), precipitation of the driest quarter that ranged from 1.2 to 2.1 cm, and an elevation that ranged from 1,500 to 2,200 m. Areas that are currently shown as >50% suitability extended from southeastern Arizona southeastward to Michoacán and Oaxaca (Figure 4).

**Figure 5:**
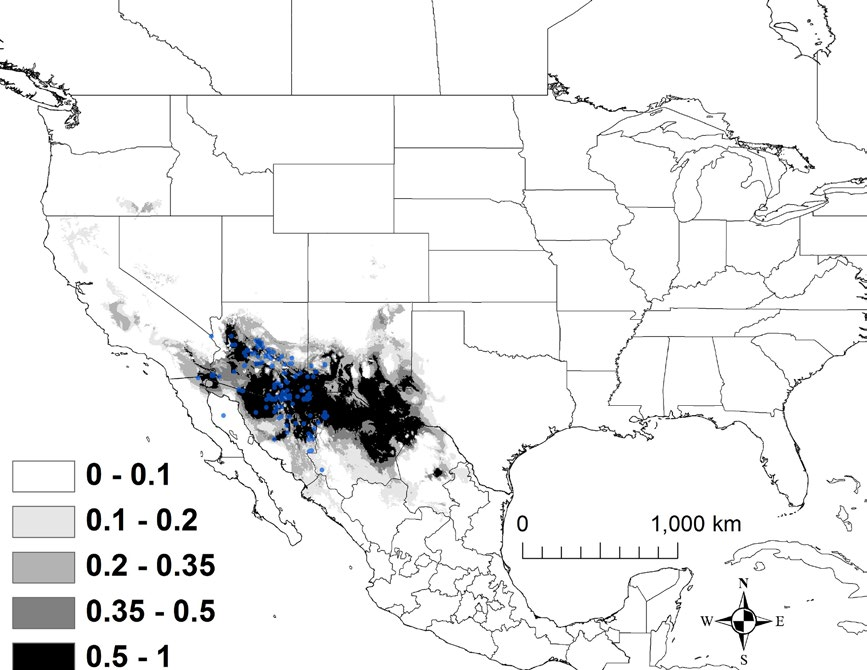
The modeled current distribution for *Kinosternon sonoriense*. The probability is shown in gray scale in the legend; the darkest shade shows an area with >0.5 probability of occurrence. Locations where *K. sonoriense* (*n = *147) were recorded as present based on EmySystem database and field observations are shown with blue dots

The best models for *K. sonoriense* included mean diurnal temperature range (BIO 2), temperature seasonality (BIO 4), mean temperature of wettest quarter (BIO 8), and precipitation of driest month (BIO 14; Table 2). The AUC for this model was 0.976 ± 0.006. There was also some model support for annual mean temperature (BIO 1), isothermality (BIO 3), maximum temperature of warmest month (BIO 5), and precipitation of driest quarter (BIO 17; Table 2). Areas that were predicted to have suitability >50% had a mean diurnal temperature range of at least 17°C, temperature seasonality from 6,000 to 7,300, and mean precipitation of the driest month of 0.3–0.8 cm. Areas that are currently shown as >50% suitable extended from New Mexico and Chihuahua west to California and Arizona (Figure 5).

**Figure 6:**
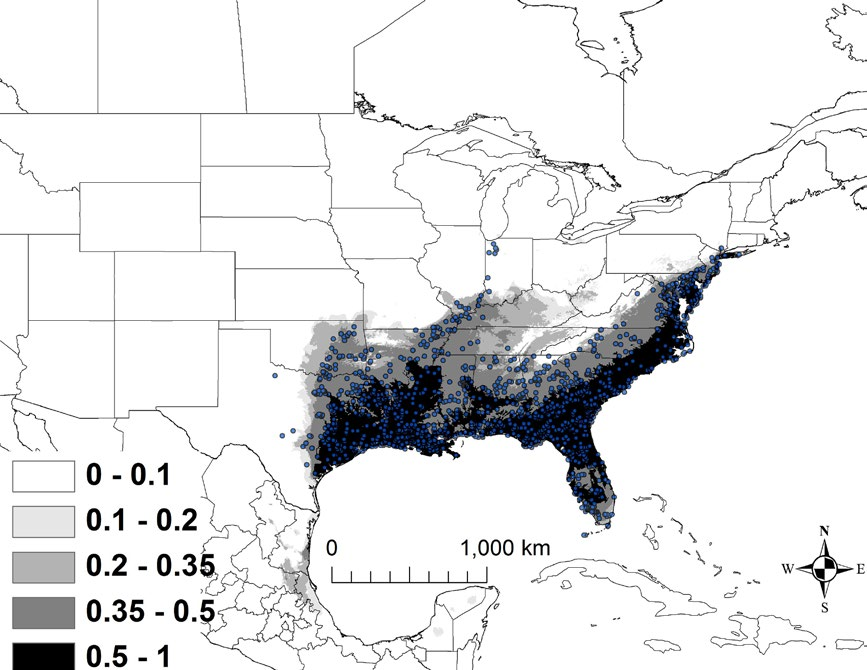
The modeled current distribution for *Kinosternon subrubrum*. The probability is shown in gray scale in the legend; the darkest shade shows an area with >0.5 probability of occurrence. Locations where *K. subrubrum* (*n = *1,472) were recorded as present based on EmySystem database and field observations are shown with blue dots

The best model for *K. subrubrum* included the variables elevation, minimum temperature of the coldest month (BIO 6), mean temperature of warmest quarter (BIO 10), and annual precipitation (BIO 12; Table 2). The AUC for this model was 0.915 ± 0.005. Areas that were predicted to have suitability >50% were less than 80 m above sea level, with a minimum temperature of the coldest month ranging from 0 to 6°C, the mean temperature of the warmest quarter ranging from 26 to 28°C, and annual precipitation ranging from 119 to 164 cm. Areas that are currently shown as >50% suitability extended from New Jersey southwest to Florida, west to Arkansas and Texas (Figure 6).

The median projected change in highly suitable conditions (i.e., those >50% suitability) for all five species was −3% (range 100%–179%), although there was considerable variation among species (Table 3). The amount of highly suitable habitat for *K. baurii* and *K. hirtipes* declined, while the amount of highly suitable habitat for *K. sonoriense* remained largely unchanged, and the amount for *K. flavescens* and *K. subrubrum* increased. However, the median amount of currently highly suitable habitat retained in future projections for these five species was only 72% (range 0%–92%).

**Table 3:** The total area predicted to have >50% probability of suitable conditions for each species under each climate change scenario

Species	Scenario	Area (km^2^)	% change in area	Area common to current (km^2^)	% current distribution retained
*Kinosternon baurii*	Current	97,886.39			
	2050—RCP 2.6	18,747.20	−80.85%	18,040.93	18.43%
	2050—RCP 4.5	15,146.41	−84.53%	14,895.92	15.22%
	2050—RCP 6.0	6,832.12	−93.02%	6,284.87	6.42%
	2050—RCP 8.5	5,330.04	−94.55%	5,125.59	5.24%
	2070—RCP 2.6	40,147.31	−58.99%	36,337.27	37.12%
	2070—RCP 4.5	6,190.85	−93.68%	5,999.67	6.13%
	2070—RCP 6.0	0.00	−100.00%	0	0.00%
	2070—RCP 8.5	0.00	−100.00%	0	0.00%
*Kinosternon flavescens*	Current	761,894.36			
	2050—RCP 2.6	1,263,789.95	65.87%	654,059.81	85.85%
	2050—RCP 4.5	1,483,646.97	94.73%	648,173.86	85.07%
	2050—RCP 6.0	1,106,486.87	45.23%	613,075.46	80.47%
	2050—RCP 8.5	1,639,647.95	115.21%	630,273.02	82.72%
	2070—RCP 2.6	1,255,586.38	64.80%	651,539.39	85.52%
	2070—RCP 4.5	1,655,794.48	117.33%	644,113.09	84.54%
	2070—RCP 6.0	1,592,850.61	109.06%	635,724.28	83.44%
	2070—RCP 8.5	2,125,826.78	179.02%	621,984.94	81.64%
*Kinosternon hirtipes*	Current	274,569.84			
	2050—RCP 2.6	249,224.98	−9.23%	191,989.18	69.92%
	2050—RCP 4.5	249,655.50	−9.07%	183,512.06	66.84%
	2050—RCP 6.0	240,300.25	−12.48%	183,320.15	66.77%
	2050—RCP 8.5	217,223.11	−20.89%	150,824.54	54.93%
	2070—RCP 2.6	230,384.60	−16.09%	180,126.63	65.60%
	2070—RCP 4.5	219,504.46	−20.06%	158,869.99	57.86%
	2070—RCP 6.0	216,845.02	−21.02%	150,506.39	54.82%
	2070—RCP 8.5	171,578.42	−37.51%	106,540.69	38.80%
*Kinosternon sonoriense*	Current	360,220.43			
	2050—RCP 2.6	342,039.32	−5.05%	278,373.89	77.28%
	2050—RCP 4.5	332,967.41	−7.57%	241,259.57	66.98%
	2050—RCP 6.0	334,081.94	−7.26%	255,422.43	70.91%
	2050—RCP 8.5	378,945.04	5.20%	274,922.19	76.32%
	2070—RCP 2.6	349,501.44	−2.98%	278,584.05	77.34%
	2070—RCP 4.5	347,657.03	−3.49%	253,839.98	70.47%
	2070—RCP 6.0	383,026.73	6.33%	264,269.59	73.36%
	2070—RCP 8.5	397,782.78	10.43%	249,467.33	69.25%
*Kinosternon subrubrum*	Current	685,299.22			
	2050—RCP 2.6	753,846.01	10.00%	624,276.89	91.10%
	2050—RCP 4.5	809,897.41	18.18%	628,949.00	91.78%
	2050—RCP 6.0	761,742.99	11.15%	628,084.40	91.65%
	2050—RCP 8.5	785,394.02	14.61%	600,940.37	87.69%
	2070—RCP 2.6	733,998.32	7.11%	604,523.99	88.21%
	2070—RCP 4.5	815,701.39	19.03%	623,163.00	90.93%
	2070—RCP 6.0	803,669.48	17.27%	613,225.66	89.48%
	2070—RCP 8.5	761,113.95	11.06%	564,144.67	82.32%

**Figure 7:**
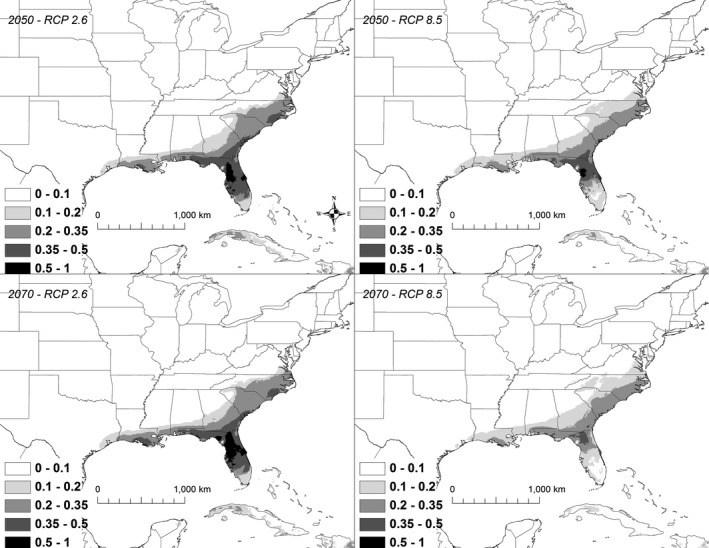
A map showing the output from the model runs for *Kinosternon baurii*. The probability of *K. baurii* occurrence is shown in gray scale in the legend; the darkest shade shows an area with >0.5 probability of occurrence

Under all scenarios, suitable conditions for *K. baurii* declined precipitously by 2050, and by 2070, highly suitable areas (i.e., those >50% suitability) largely disappeared (Figure 7). A total of 97,886 km^2^ was identified as being currently highly suitable (i.e., >50% chance of suitable conditions). By 2050, the amount of highly suitable habitat declined to 5,330–18,747 km^2^, of which only 5%–18% was shared with the current model (Table 3). By 2070, the amount of highly suitable habitat declined to 0–40,147 km^2^, of which 0%–37% was shared with the current model (Table 3).

**Figure 8:**
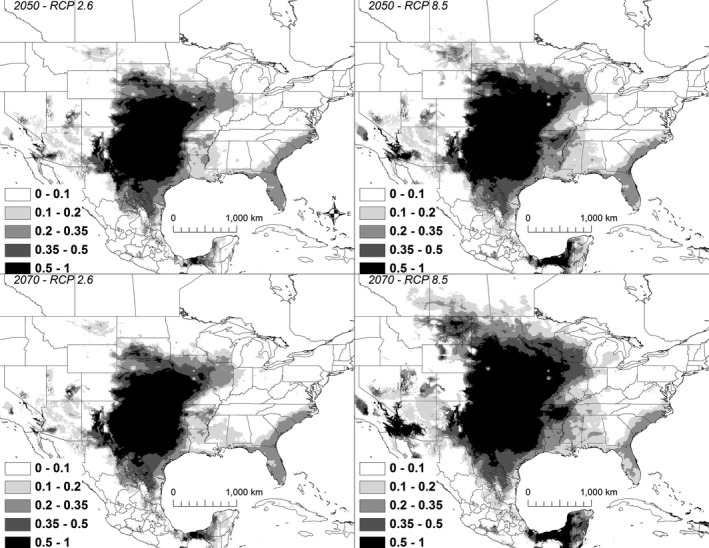
A map showing the output from the model runs for *Kinosternon flavescens*. The probability of *K. flavescens* occurrence is shown in gray scale in the legend; the darkest shade shows an area with >0.5 probability of occurrence

Under all scenarios, highly suitable conditions (i.e., suitability >50%) for *K. flavescens* extended north to South Dakota by 2050 and with some scenarios showing suitable conditions in Montana by 2070 (Figure 8). A total of 761,894** **km^2^ was identified as being currently highly suitable. By 2050, the amount of suitable habitat increased substantially, ranging from 1,106,487 to 1,639,647 km^2^ (Table 3). However, only 80%–86% of the highly suitable habitat during 2050 was shared with the current model (Table 3). By 2070, the amount of highly suitable habitat increased ranging from 1,255,586 to 2,125,826 km^2^, of which 82%–85% was shared with the current model (Table 2).

The mean current modeled suitability for *K. flavescens* within Illinois, Iowa, and Missouri, where the species is currently threatened with extirpation (Christiansen et al., 2012), was 12% and ranged from 0% to 50%. By 2050, the suitability of this region increased. For 2050, mean suitability increased from a mean of 30% (range 0%–69%) under the RCP 2.6 scenario, to a mean suitability of 40% (range 3%–77%) under the RCP 8.5 scenario. Suitability in these three states was even higher by 2070 under some scenarios. Mean suitability under the 2070 RCP 2.6 scenario was still 30% (range 0%–69%), but mean suitability under the 2070 RCP 8.5 scenario increased to 48% (range 3%–78%).

**Figure 9:**
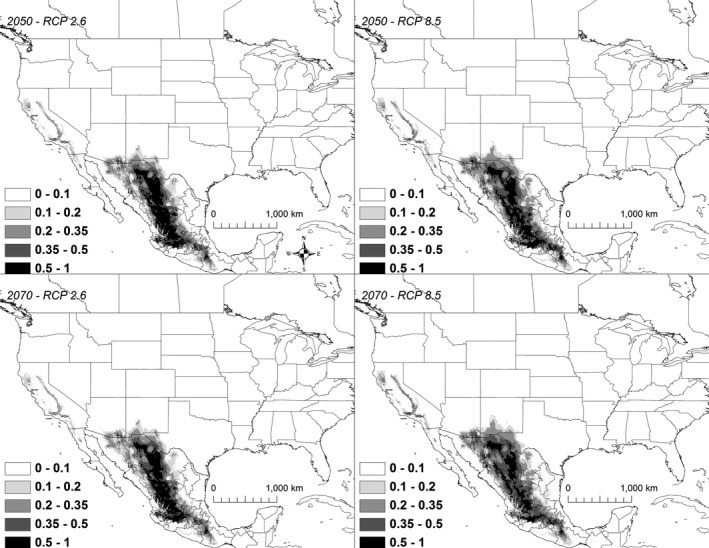
A map showing the output from the model runs for *Kinosternon hirtipes*. The probability of *K. hirtipes* occurrence is shown in gray scale in the legend; the darkest shade shows an area with >0.5 probability of occurrence

Under all scenarios, highly suitable conditions (i.e., >50%) for *K. hirtipes* shifted minimally north‐northwest and the amount of highly suitable habitat declined (Figure 9). A total of 274,570 km^2^ was identified as being currently highly suitable. By 2050, the amount of suitable habitat declined moderately, ranging from 217,223 to 249,656 km^2^ (Table 3). However, only 55%–70% of the highly suitable habitat during 2050 was shared with the current model (Table 3). By 2070, the amount of highly suitable habitat declined further, ranging from 171,578 to 230,385** **km^2^, of which only 39%–66% was shared with the current model (Table 3).

**Figure 10:**
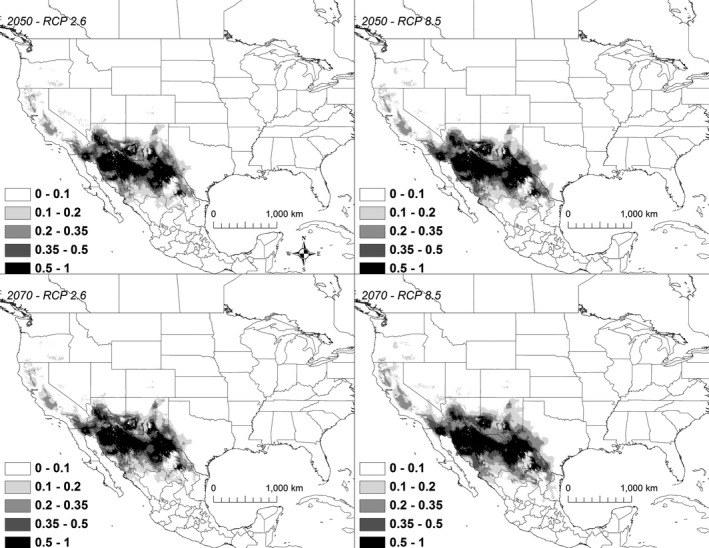
A map showing the output from the model runs for *Kinosternon sonoriense*. The probability of *K. sonoriense* occurrence is shown in gray scale in the legend; the darkest shade shows an area with >0.5 probability of occurrence

Under nearly all scenarios, highly suitable habitat for *K*. *sonoriense* shifted slightly south, although under the RCP 2.6 2050 scenario, there was a slight westward shift (Figure 10). A total of 360,224 km^2^ was identified as being currently highly suitable. Under three of the four 2050 scenarios, the amount of highly suitable habitat decreased, ranging from 332,967 to 342,039 km^2^, of which 67%–77% was shared with the current model (Table 3). However, under the 2050 RCP 8.5 scenario, the amount of highly suitable habitat increased to 378,945 km^2^, of which 76% was shared with the current range (Table 3). By 2070, two models (RCP 2.6 and RCP 4.5) projected a slight decline of approximately 3% in the amount of potentially suitable habitat, while under the RCP 6.0 scenario, suitable habitat increased by 6% and under the RCP 8.5 scenario, suitable habitat was expected to increase by 10%. However, by 2070, only 69%–77% of the highly suitable habitat was shared with the current model (Table 3).

**Figure 11:**
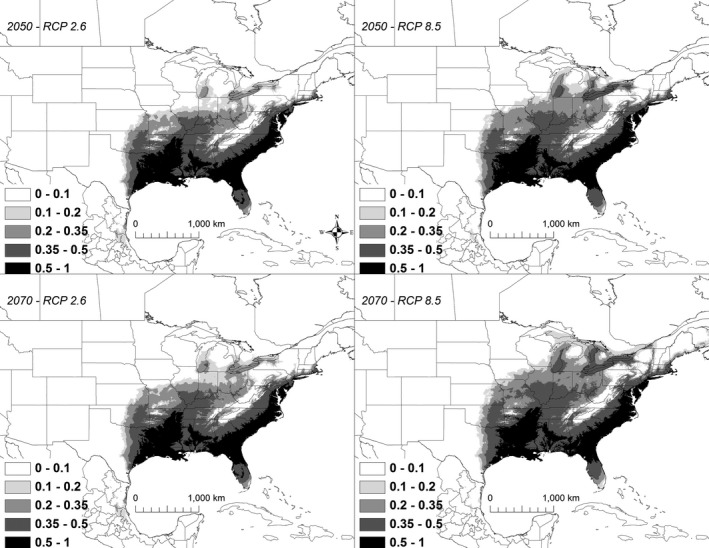
A map showing the output from the model runs for *Kinosternon subrubrum*. The probability of *K. subrubrum* occurrence is shown in gray scale in the legend; the darkest shade shows an area with >0.5 probability of occurrence

Under all scenarios, suitable conditions for *K. subrubrum* expanded to the northeast (Figure 11). A total of 685,299 km^2^ was identified as being currently highly suitable. Under all 2050 scenarios, the amount of highly suitable habitat increased by approximately 10%–18% relative to the current model, ranging from 753,846 to 809,897 km^2^, of which 88%–92% was shared with the current model (Table 3). By 2070, the amount of highly suitable habitat ranged from 733,998 to 815,701 km^2^, of which 88%–91% was shared with the current model (Table 3).

**Figure 12:**
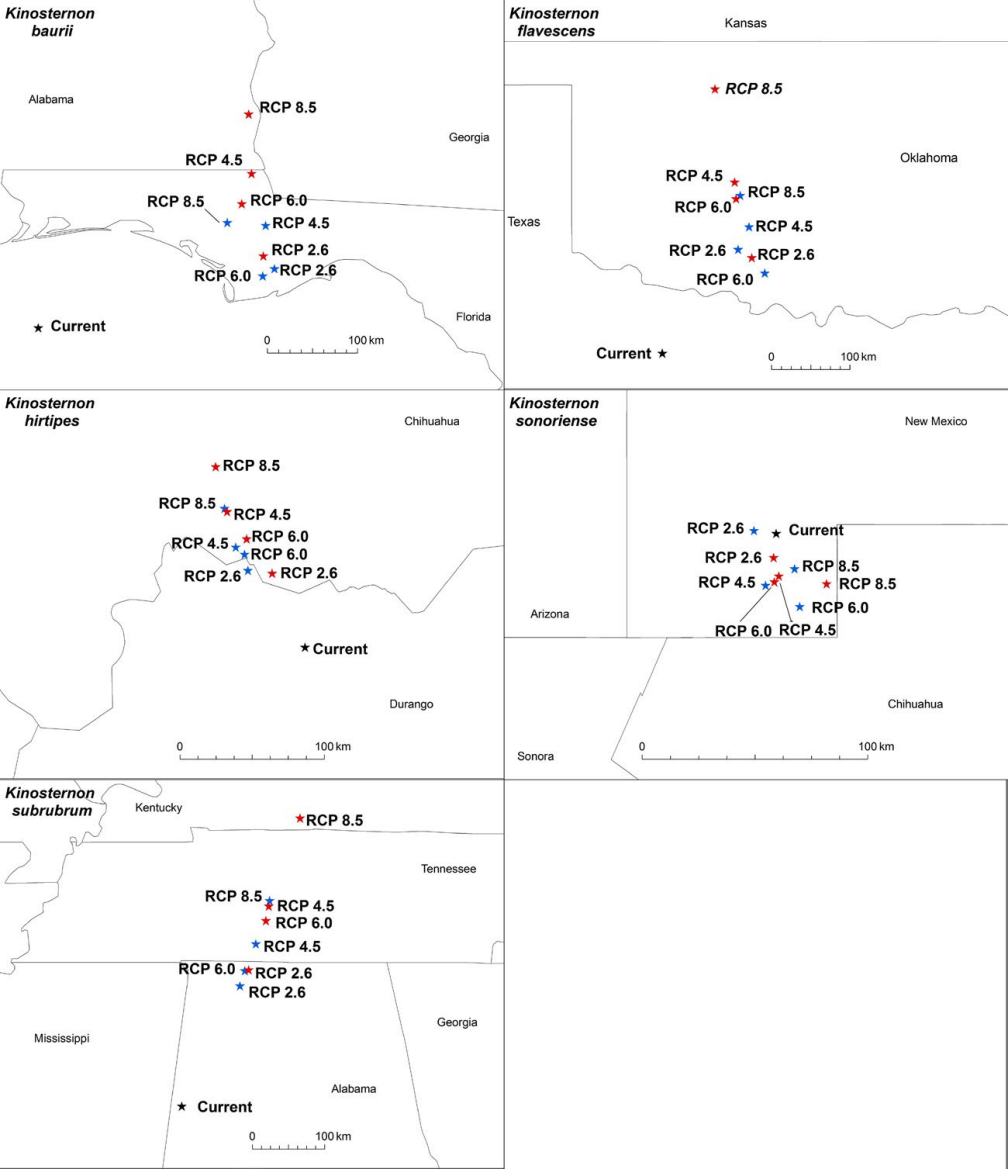
The centroids (stars) showing the geometric center of the distribution under each scenario for each species. The current centroid is shown with a black star, while projected centroids by 2050 are shown with blue stars and projected centroids by 2070 are shown with red stars. Due to the concave distribution of *Kinosternon baurii*, the current centroid is located in the Gulf of Mexico, where *K. baurii* is not expected to occur

For four of the five species considered, centroids shifted generally northward (Figure 12). The only exception was *K. sonoriense*, where the centroids shifted short distances to the south (Figure 12). The median projected centroid shift for these five species was 37.5 km per decade, but considerable species‐specific variability exists in the response rate. Under all scenarios, the rate of change for *K. sonoriense* was only 2–8 km per decade (Table 4). Centroids for *K. hirtipes* shifted at a moderate rate of 9–27 km per decade. In contrast, the rate of change was much faster for *K. flavescens* centroids (26–57 km per decade) and for *K. subrubrum* centroids (34–75 km per decade). The apparent large shift to the northeast for *K. baurii* centroids (39–62 km per decade) should be viewed with caution, however, as the amount of suitable habitat for this species is projected to rapidly decline, resulting in greater weights given to areas currently outside the known distribution of this species.

**Table 4:** A summary of the distance from each centroid for each scenario to the current centroid and the rate per decade

Species	Scenario	Distance (km) and direction to current	Rate per decade
*Kinosternon baurii*	2050—RCP 2.6	237 (ENE)	59 km/decade
	2050—RCP 4.5	247 (ENE)	62 km/decade
	2050—RCP 6.0	224 (ENE)	56 km/decade
	2050—RCP 8.5	216 (NE)	54 km/decade
	2070—RCP 2.6	231 (NE)	39 km/decade
	2070—RCP 4.5	267 (NE)	44 km/decade
	2070—RCP 6.0	239 (NE)	40 km/decade
	2070—RCP 8.5	311 (NE)	52 km/decade
*Kinosternon flavescens*	2050—RCP 2.6	154 (NE)	39 km/decade
	2050—RCP 4.5	185 (NE)	46 km/decade
	2050—RCP 6.0	149 (NE)	37 km/decade
	2050—RCP 8.5	217 (NNE)	54 km/decade
	2070—RCP 2.6	154 (NE)	26 km/decade
	2070—RCP 4.5	231 (NNE)	38 km/decade
	2070—RCP 6.0	211 (NNE)	35 km/decade
	2070—RCP 8.5	341 (NNE)	57 km/decade
*Kinosternon hirtipes*	2050—RCP 2.6	63 (NNW)	16 km/decade
	2050—RCP 4.5	81 (NNW)	20 km/decade
	2050—RCP 6.0	74 (NNW)	18 km/decade
	2050—RCP 8.5	108 (NNW)	27 km/decade
	2070—RCP 2.6	55 (NNW)	9 km/decade
	2070—RCP 4.5	105 (NNW)	17 km/decade
	2070—RCP 6.0	83 (NNW)	14 km/decade
	2070—RCP 8.5	136 (NNW)	23 km/decade
*Kinosternon sonoriense*	2050—RCP 2.6	8 (W)	2 km/decade
	2050—RCP 4.5	23 (S)	6 km/decade
	2050—RCP 6.0	34 (SSE)	8 km/decade
	2050—RCP 8.5	17 (SSE)	4 km/decade
	2070—RCP 2.6	11 (S)	2 km/decade
	2070—RCP 4.5	19 (S)	3 km/decade
	2070—RCP 6.0	21 (S)	4 km/decade
	2070—RCP 8.5	23 (SE)	5 km/decade
*Kinosternon subrubrum*	2050—RCP 2.6	179 (NNE)	45 km/decade
	2050—RCP 4.5	240 (NNE)	60 km/decade
	2050—RCP 6.0	201 (NNE)	50 km/decade
	2050—RCP 8.5	302 (NNE)	75 km/decade
	2070—RCP 2.6	203 (NNE)	34 km/decade
	2070—RCP 4.5	294 (NNE)	49 km/decade
	2070—RCP 6.0	274 (NNE)	46 km/decade
	2070—RCP 8.5	421 (NNE)	70 km/decade

## 4 Discussion

Maxent was effective at predicting the actual current distributions of all five species. In general, the highest probability of occurrence for each species was in the core of the species geographic ranges, with decreasing probabilities as the edge of a species geographic range was approached. In some cases, moderately suitable habitat (<50% suitability) outside the geographic range of a species was projected to occur. For example, models for both *K. sonoriense* and *K. hirtipes* predicted moderately suitable habitat in the Central Valley of California. As Maxent can be used to predict the distribution of turtles in novel habitats (Ficetola et al., 2009), and as multiple non‐native turtle species are established in California (Jennings, 1983; Thomson, Spinks, & Shaffer, 2010), it is not surprising that potentially suitable habitat was identified outside of core ranges. The Mojave Desert is between the current distributions of these *Kinosternon* species and central California. This desert acts as a biogeographical barrier, separating potentially suitable habitat from the area already occupied by these species. Consequently, it is also not surprising that *K. sonoriense* and *K. hirtipes* have not colonized this region, although historical records of *K. sonoriense* in the Colorado River drainage are evidence that *K. sonoriense* has dispersed right up to the edge of the Mojave Desert barrier (Ernst & Lovich, 2009).

Turtles, and other ectotherms, are thought to be particularly sensitive to climate changes (Barrows, 2011; Diamond et al., 2012; Duarte et al., 2012; Gibbons et al., 2000; McCoy et al., 2011; Rödder et al., 2013). Our models suggest that highly suitable habitat for *K. baurii* and *K. hirtipes* will decline in the coming decades, while highly suitable habitat will remain essentially unchanged for *K. sonoriense* and will increase for *K. flavescens* and *K. subrubrum*. These results broadly mirror the results reported in Ihlow et al. (2012) although they predicted that the range of *K. hirtipes* and *K. sonoriense* would increase instead of declining and remaining unchanged, respectively. These differences may be due to differences in sample size, resolution, or the climate change scenarios examined.

However, even when the range of the species is predicted to increase, the chelonian may not be able to expand its range in concordance with the shift in suitable habitat. For example, only 80%–86% of the currently highly suitable habitat for *K. flavescens* will still be highly suitable by 2050 although newly created highly suitable habitat will occur to the north of this area. Consequently, even though the total amount of highly suitable habitat for this species is expected to double by 2050, it is expected that there will be a lag, and possibly even a decrease in the population, before *K. flavescens* can expand to occupy potentially suitable areas.

A precipitous decline in the extent of highly suitable conditions for *K. baurii* is expected under all scenarios. *K. baurii* is currently a relatively common turtle throughout most of Florida (Einem, 1956; IUCN 2011) with the notable exception of the Lower Keys population (IUCN 2011). However, our models suggested that the amount of highly suitable habitat in the southeastern United States would decline by 81%–95% by 2050. These results mirrored those reported by Ihlow et al. (2012) who suggested that the range of *K. baurii* would virtually disappear in the coming decades. While turtles can exhibit behavioral plasticity in response to suboptimal climate (Lovich et al., 2012; Millar, Graham, & Blouin‐Demers, 2012; Refsnider & Janzen, 2012), it is unlikely that these adaptations will suffice indefinitely. It seems more likely that populations of *K. baurii* will decline substantially during the 21st century in response to climate change.

In contrast to *K. baurii*, the area of potentially suitable climate for *K. flavescens* is expected to approximately double by 2050 (Table 3). Ihlow et al. (2012) likewise predicted an increase in the distribution of these species under all scenarios. Although the IUCN (2011) lists *K. flavescens* as a species of Least Concern, Christiansen et al. (2012) documented recent severe declines in isolated populations in Illinois, Iowa, and Missouri. These declines are thought to be due primarily to a combination of predation and habitat loss (Christiansen et al., 2012). However, Christiansen et al. (2012) also noted that these small, isolated populations may be susceptible to temperature extremes. If populations can be maintained in these states in the coming decades, temperature extremes may be less of a concern. According to our models, mean suitability in these three states is currently 12% but is expected to rise to 30%–40% by 2050.

The centroid for each species shifted at a median rate of 37.5 km per decade across all scenarios (range 2–75 km/decade). However, *Kinosternon* species do not typically exhibit great vagility. For example, although *K. subrubrum* may travel more than 1 km through water (Cordero, Reeves, & Swarth, 2012), and *K. sonoriense* has been recorded dispersing up to 7.2 km between captures (Hall & Steidl, 2007), movements of more than 500 m are rare (Cordero et al., 2012; Hall & Steidl, 2007). In addition, barriers to dispersal, both natural (e.g., deserts, large rivers) and human‐induced (e.g., roads, drained wetlands), may also inhibit the spread of *Kinosternon* species in response to climate change. Furthermore, our models did not account for the forecast sea level rise of 0.5–1.4 m above 1990 levels by the end of the 21st century (Rahmstorf, 2007). Finally, our simulations model the potential changes in climatic suitability but did not attempt to model changes in land use, which may present even greater hurdles for the persistence of turtles in the future. For example, fire suppression in Iowa, Illinois, and Missouri may lead to vegetative community succession and reduce the amount of open habitat available for *Kinosternon flavescens* for overwintering and nesting. It seems unlikely that *Kinosternon* spp. will be able to shift their distributions rapidly enough to keep pace with climate change and land use changes.

In addition, changes in temperature may also skew sex ratios as *Kinosternon* species exhibit temperature‐dependent sex determination, with higher temperatures resulting in more females (Vogt & Bull, 1982). Mitchell and Janzen (2010) noted that reptiles have apparently adapted to previous climate change events but also noted that the amount of warming forecasted for the 21st century is unusually rapid. Efforts to reduce nest temperatures by shading nests were successful in increasing the proportion of male hatchling turtles in one study (Patino‐Martinez et al., 2012), but this method is unlikely to be effective beyond the local scale. Refsnider and Janzen (2012) suggested that freshwater turtles may exhibit behavioral plasticity with regard to shade cover for nest sites, and females may be able to adjust the timing of nesting season to compensate to some degree for future warmer climates (Mazaris, Kallimanis, Pantis, & Hays, 2013). However, Mitchell and Janzen (2010) suggested that increasing the number of females in a population as a result of climate change will be unlikely to cause the population to fail as long as there are still some males present.

Lavergne et al. (2010) note that organisms may not be able to adapt to climate change if the rate of change is too rapid and the demography is not sufficiently dynamic. Chelonians in general exhibit long generation times and high adult survivorship coupled with high juvenile mortality. The annual survival rate for adult *Kinosternon* species approaches or exceeds 90% (Frazer, Gibbons, & Greene, 1991; Iverson, 1991). These chelonians also exhibit a variety of behavioral and physiological methods for dealing with unfavorable conditions (Iverson, 1991; Ligon & Stone, 2003). Consequently, although conditions may become increasingly unsuitable in parts of their range due to climate change, local extinction may be delayed in response (Jackson et al., 2001). However, this extinction debt (sensú Jackson & Sax, 2010) will only delay local extinctions, not preclude them.

## Conflict of Interest

None declared.
